# Effects of Pediatric Rehabilitation on Children With Spastic Quadriplegia Primary to Seizure Disorder and Global Developmental Delay: A Case Report

**DOI:** 10.7759/cureus.56189

**Published:** 2024-03-14

**Authors:** Neha M Chitlange, H V Sharath, Akshaya Saklecha, Sakshi Desai

**Affiliations:** 1 Department of Pediatric Physiotherapy, Ravi Nair Physiotherapy College, Datta Meghe Institute of Higher Education and Research (DU), Wardha, IND; 2 Department of Neurophysiotherapy, Ravi Nair Physiotherapy College, Datta Meghe Institute of Higher Education and Research (DU), Wardha, IND

**Keywords:** spastic quadriplegia, seizure disorder, global developmental delay, pediatric physiotherapy, rehabilitation

## Abstract

The most severe form of spastic cerebral palsy (CP), which affects the arms and legs and often the face, is known as spastic quadriplegia. In addition to other developmental disabilities such as intellectual disability and seizures, it can cause difficulty in walking. Children with CP often have seizures as a result of brain injury, and spastic quadriplegic CP is typically associated with global developmental delay. For the purpose of addressing the unique motor and functional challenges associated with spastic quadriplegia, neurophysiotherapy is essential. This treatment includes neurodevelopmental techniques, posture and balance training, and activities aimed at improving gait. The purpose of this case study is to demonstrate how early and continuous physical therapy interventions can maximize a child's functional abilities and prevent further complications. In this instance, a five-year-old boy with a documented history of spastic quadriplegia, seizure disorder, and global developmental delay reported experiencing challenges with sitting, walking, and speech. He had three episodes of fever, which led to his hospital admission. The child's medical history included acute hemorrhagic encephalitis, mild hydroureteronephrosis on the left side, and persistent convulsions that affected only one side of the body. Bilateral thalamic altered signal intensities were observed in the brain's MRI, and multiple calcifications were detected in the periventricular cortex, thalamus, and basal ganglia on the brain's CT scan. To enhance the independence, strength, and coordination of voluntary movement in individuals with CP, a variety of techniques are used in addition to physical therapy, such as occupational therapy, speech therapy, aquatic therapy, constraint-induced movement therapy, functional electrical stimulation, orthotic devices, injections of botulinum toxin, and hippotherapy.

## Introduction

Children with cerebral palsy (CP) are most frequently affected by neuromotor disabilities, but the underlying cause is often still unidentified [1]. Spastic quadriplegia, which accounts for 5% of all cases of CP, is characterized by equal impairment in all limbs, particularly the upper limbs [2]. Both fine and gross motor skills are affected by spastic quadriplegia, albeit to differing degrees in their affected patients. The most severe form of spastic CP, known as quadriplegia, is characterized by bipyramidal syndrome and severe mental retardation [3]. People who suffer from spastic quadriplegia frequently link to other conditions like movement disorders and sensory disorders [4]. There are between 25% and 80% of children with spastic quadriplegia who also have comorbid conditions like seizures, malnourishment, gastrointestinal issues, and sensory disturbances. In developing and impoverished nations, spastic quadriplegia is more common. Prematurity (birth before 37 weeks of pregnancy) and acute fetal distress during pregnancy and delivery, as indicated by a low APGAR score, are the risk factors for spastic quadriplegia [5]. Risk factors that can cause spastic quadriplegia can be prenatal, perinatal, and postnatal. Genetic factors specifically play a role between these primary prenatal factors and the majority of cases of spastic paralysis [5]. Intrapartum perinatal factors are those that operate from the beginning of labor to the delivery. When an infant or toddler's nervous system is still developing, postnatal causes, also known as postpartum, can manifest either soon after birth or later [5].

Children with CP are more prone to experience seizures and delayed motor skill development. Seizures are more common in children with CP due to brain injuries that occur before, during, or shortly after birth and result in abnormal nerve activity in the brain. Their shared global developmental delay hinders their capacity to move, think, speak, and feel emotions [6]. Global developmental delay and seizure disorder coexist in CP, which is a significant challenge that frequently necessitates medical care, including prescription medication, dietary modifications, and, in severe cases, surgery [5]. Anticonvulsant drugs are widely used in the treatment of seizures in children with CP. It is crucial to recognize developmental delays and seizures as soon as possible in order to provide timely diagnosis and treatment [7].

Physical therapy is a vital component of CP management, and nearly every individual with a CP diagnosis receives PT services. Physiotherapy aims to lessen the physical symptoms of the condition and support the child with CP in meeting their participation needs. To improve the independence, power, and coordination of voluntary movements, physiotherapists employ a variety of therapeutic approaches [8]. An increasing number of therapies have been studied in the field of physiotherapy for CP [9]. By reducing the impact of their physical limitations, pediatric rehabilitation enhances the quality of life for children with CP and their families while assisting them in realizing their full potential in terms of physical independence and fitness [9,10].

## Case presentation

A five-year-old male child with a known case of spastic quadriplegia with seizure disorder and a global developmental delay came with complaints of difficulty in sitting, inability to walk and talk, and a history of three episodes of seizures and got admitted to Acharya Vinobha Bhave Rural Hospital (AVBRH) for further management. As narrated by the mother, the child was apparently alright till four months of age. Then, the child had one episode of fever followed by convulsions, wherein he was taken to a local hospital and was admitted for 15 days and was diagnosed with acute hemorrhagic encephalitis with left-sided mild hydroureteronephrosis. She gave a history that the baby cried five minutes after the full-term normal delivery. He was again admitted to the hospital for 15 days in January 2022 as the patient had frequent episodes of chronic type of convulsions involving one side of the body. MRI of the brain was done and was suggestive of (s/o) altered signal intensities involving the bilateral thalamus and suggestive of hemorrhagic encephalitis. CT brain revealed multiple calcifications in the bilateral basal ganglia, the thalamus, and the periventricular cortex. For the above complaint, the patient was prescribed folic acid tablet (Tab), baclofen, and valproic acid syrup (Syp).

At five years of age, the child had one episode of convulsion for which he was admitted to Yavatmal Government Hospital and was started on levetricetam (Syp) and valproic acid (Syp). In November, the child was admitted to Yavatmal with a complaint of fever for two days. Contrast-enhanced MRI brain was done which was suggestive of subtle nonenhancing hyperintensity noted along the periventricular region and the thalamus secondary to perinatal insult and was shifted on valparin (Syp), levipil (Syp), pacitane (Tab), and baclofen antibiotics given for five days. And now, the child has been brought to AVBRH for further management. Physiotherapy was started on the 2nd day of admission. Gross motor, fine motor, social, and language milestones are given in Tables 1-4. The child has never been able to perform any of the milestones marked as NA (not attained).

**Table 1:** Gross motor milestones NA: Not attained

Milestones	Normal	Month attained
Neck holding	3 months	NA
Roll over	4 months	3 years
Sits with support	6 months	NA
Sitting without support	8 months	NA
Stands with support	9 months	NA
Stands without support	12 months	NA
Walks alone, creeps upstairs	15 months	NA
Runs and explore drawers.	18 months	NA
Walks up and downstairs (2 feet/step)	2 years	NA
Walks up and downstairs (alternate feet)	3 years	NA

**Table 2:** Fine motor milestones NA: Not attained

Milestones	Normal	Month attained
Bidextrous reach	4 months	3 years
Unidextrous reach	6 months	4 years
Immature pincer grasp	9 months	4 ½ years
Mature pincer grasp	12 months	NA
Imitates scribbling	5 months	NA
Scribbles	18 months	NA
Vertical and circular strokes	2 years	NA
Copies circle	3 years	NA
Copies cross	4 years	NA
Copies triangle	5 years	NA

**Table 3:** Social milestones NA: Not attained

Milestones	Normal	Month attained
Social smile	2 months	NA
Recognises mother	3 months	3 years
Stranger anxiety	6 months	NA
Waves bye bye	9 months	4 years
Comes when called	12 months	NA
Copies parents in task	18 months	NA
Asks for food, drink	2 years	3 years.
Knows full name and gender	3 years	NA
Plays cooperatively in group	4 years	NA
Dresses and undresses	5 years	NA

**Table 4:** Language milestones NA: Not attained

Milestones	Normal	Month attained
Alerts to sounds	1 month	2 years
Coos	3 months	2 years
Laugh loud	4 months	2 years
Monosyllables	6 months	4 years
Bisyllables	9 months	NA
1-2 words with meaning	12 months	NA
8-10 vocabulary	18 months	NA
2-3-word sentences	2 years	NA
Knows name and gender, asks questions	3 years	NA
Tells stories, poem	4 years	NA
Asks the meaning of words	5 years	NA

Clinical findings

The patient was conscious and oriented to person. Verbal consent was obtained from his mother before an examination was performed. His speech was affected, but his hearing and vision were unaffected. He was assessed in a supine lying position. On the day of the examination, his Glasgow Coma Scale score was 13/15 (E4V4M5). The patient was afebrile, i.e., 37°C, his respiratory rate was 24 breaths/minute, his pulse rate was 98 beats/minute, and he was able to sustain oxygen saturation in room air. His height and weight were 97 cm and 11 kg, respectively. His head circumference was 47 cm. On neurological examination, sensations were intact. Table 5 shows the muscle tone (Modified Ashworth Scale) of the patient's upper and lower extremities. Global hyperreflexia (+++) was seen in the deep tendon reflexes.

**Table 5:** Muscle tone of the upper and lower extremities of motion 1+: slight increase in muscle tone, manifested by a catch and release or by minimal resistance throughout the remainder (less than half) of the range

Muscle tone	Right side	Left side
Biceps	1+	1+
Triceps	1+	1+
Muscles of forearm and hand	1+	1+
Quadriceps muscles	1+	1+
Calf muscles	1+	1+

Medical management

The medical management of children with spastic quadriplegia primary to seizure disorder and global developmental delay necessitates a comprehensive approach aimed at addressing both the underlying neurological conditions and associated symptoms. This involves a tailored regimen of antiepileptic medications to manage seizures effectively, coupled with therapies such as physical, occupational, and speech therapy to optimize motor function, communication skills, and overall development. Additionally, close monitoring for potential medication side effects and periodic reassessment of the treatment plan are crucial for ensuring optimal outcomes and quality of life for these children. Medication with it's dosage is given in Table 6.

**Table 6:** Medication with its dosage ml: milliliter; mg: milligram; BD: bis in die (twice a day) ; OD: once daily

Medication	Dosage	Duration
1. Valproic acid (syrup)	5 ml	BD
2. Levetiracetam (syrup)	2.5 ml	BD
3. Baclofen (tablet)	10 mg	OD

Rehabilitation management

The rehabilitation management of children with spastic quadriplegia primary to seizure disorder and global developmental delay, a multidisciplinary approach was employed to address the complex needs of the patients. The management strategy integrated various therapeutic modalities including physiotherapy, speech therapy, and pharmacological interventions aimed at seizure control and addressing developmental delays. Individualized rehabilitation plans were formulated based on the specific needs and functional goals of each child, with an emphasis on maximizing functional independence and quality of life. The results of the intervention demonstrated significant improvements in motor function, communication skills, and overall independence in daily activities among the children. Furthermore, the collaborative efforts of the medical and rehabilitation teams facilitated early detection and management of complications, improved seizure control, and optimized developmental outcomes. This case report underscores the importance of a comprehensive and coordinated approach to pediatric rehabilitation in children with complex neurological conditions, highlighting the potential for significant functional gains and improved quality of life with appropriate interventions given in Table 7. Improvement (outcome measures) of intervention is seen in Table 8.

**Table 7:** Rehabilitation protocol Source: [11]

Problem list	Physiotherapy goals	Therapeutic intervention
Lack of parent’s motivation	To educate the parents	To set reasonable expectations and do exercises at home
Lack of motivation and involvement of the child in sessions	To involve the child in sessions	Engage in 20 minutes of ball-playing activities
Spasticity	To normalize the muscle tone in hypertonic muscle groups	Rood's inhibitory techniques includes deep tendinous pressure, prolonged icing for 20 minutes, sustained stretching for 30 seconds with three reps. The application of slow and rhythmic proprioceptive training was conducted on the upper, lower, and trunk limbs from semi-reclined and quadruped positions [11]. Rhythmic rotations
Unable to control head and on forearms posture	To initiate symmetrical head raising and upright holding, raise your head and maintain a straight posture and to facilitate head raise and turn	Neurodevelopmental techniques includes head control facilitation involves raising the head, maintaining postural stability, and counterpoising movement by turning the head side to side
Postural instability	To improve postural mechanisms	Therapeutic balls are used to facilitate righting and equilibrium reactions by tilting at different directions. Counterposing exercises
Unable to sit without support and stand	To facilitate trunk control	Supine transition to sitting. Altering from standing to sitting position. Quadruped rocking and reaching activities, crawling, sliding with support, trunk control on Swiss ball. Chair swings, rocking horse. Standing frames-arm reach. Perturbations of a child in standing. Standing frame-supported wheel chair
Contracture prevention	To decrease the contracture in the lower limb	Static weight-bearing exercises using standing frames

**Table 8:** Outcome measures GMFCS: Gross Motor Functional Classification Scale

Scale	Pretreatment score	Posttreatment score (3-month follow-up)
Functional Mobility Scale	Level 2	Level 1
GMFCS	Level 5	Level 5
Wee Functional Independence Measure	34/126	54/126
Manual Ability Classification System	Level 2	Level 1

Outcome measures

The Functional Mobility Scale (FMS) is a parent-reported measure of a child's mobility in various natural settings, assessing the need for assistive devices like walkers, crutches, or wheelchairs in different environments such as home, school, and community. It consists of six levels based on the usual need for assistive devices and is designed to evaluate functional mobility in children and adolescents with CP. The Gross Motor Function Classification System (GMFCS) categorizes the mobility and gross motor skills of individuals with CP into five levels, providing a clear description of current motor function and potential future needs for mobility aids like crutches, walkers, or wheelchairs. The Wee Functional Independence Measure (WeeFIM) is an 18-item, seven-level ordinal scale instrument that measures a child's consistent performance in essential daily functional skills, including self-care, mobility, and cognition. It is based on the format of the Functional Independence Measure (FIM) for adults and is designed to assess functional independence in children and adolescents with CP and other developmental disabilities. The WeeFIM is categorized into two main functional streams: "Dependent" (requires helper: scores 1-5) and "Independent" (requires no helper: scores 6-7). Scores 1 (total assistance) and 2 (maximal assistance) belong to the "Complete Dependence" category, while scores 3 (moderate assistance), 4 (minimal contact assistance), 5 (supervision or set-up), 6 (modified independence), and 7 (complete independence) belong to the "Modified Dependence" and "Independent" categories. The Manual Ability Classification System (MACS) is a tool used to classify the manual abilities of children with CP based on their self-initiated ability to handle objects in daily activities. The MACS consists of five levels, ranging from Level I (able to handle objects easily and successfully) to Level V (limited ability to handle objects).

## Discussion

In the case report detailing the effect of pediatric rehabilitation on children with spastic quadriplegia primary to seizure disorder and global developmental delay, several key observations emerge. Firstly, the interdisciplinary approach of pediatric rehabilitation proves instrumental in addressing the multifaceted needs of these children. Through a combination of physical, occupational, and speech therapy, tailored interventions are designed to enhance motor skills, communication abilities, and overall functional independence. Such comprehensive care not only targets specific impairments but also fosters holistic development, thereby maximizing the potential for improved outcomes [12-14].

Furthermore, the significance of early intervention cannot be overstated in mitigating the impact of spastic quadriplegia, seizure disorder, and global developmental delay. By initiating rehabilitation strategies during the critical developmental stages, clinicians can capitalize on the brain's plasticity and facilitate neural reorganization. This proactive approach aims to minimize secondary complications, optimize motor function, and promote adaptive behaviors, thereby laying a solid foundation for long-term progress and quality of life.

The observed improvements in motor function, communication skills, and activities of daily living underscore the transformative impact of pediatric rehabilitation on the child's functional abilities and quality of life. Through targeted interventions aimed at improving muscle tone, coordination, and mobility, the child achieved significant milestones, enhancing participation in social interactions, play activities, and self-care tasks. Such advancements not only empower the child but also alleviate caregiver burden and enhance family dynamics [15]. The plasticity of the developing brain plays a pivotal role in driving neurodevelopmental progress, particularly in children with neurological impairments. Pediatric rehabilitation exploits the principles of neuroplasticity through structured interventions that stimulate adaptive changes in neural circuits, facilitating motor learning, cognitive processing, and sensory integration. This case report underscores the remarkable potential for functional gains even in children with complex neurodevelopmental conditions, highlighting the critical window of opportunity for intervention [16].

Family engagement and empowerment are integral components of pediatric rehabilitation, as caregivers play a central role in facilitating the child's progress and well-being. The collaborative partnership between rehabilitation professionals and families fosters a supportive environment conducive to holistic care planning, goal setting, and skill development. Moreover, providing caregivers with education, training, and psychosocial support enhances their capacity to advocate for their child's needs and navigate the challenges associated with caregiving. Advancements in technology offer promising avenues for enhancing pediatric rehabilitation outcomes, particularly in harnessing virtual reality, robotics, and assistive devices to augment traditional therapeutic approaches. Integrating innovative technologies into rehabilitation protocols can enhance engagement, motivation, and intensity of therapy, thereby maximizing functional gains and promoting neuroplasticity. However, ensuring equitable access to technology and addressing potential barriers to implementation are crucial considerations [17-20].

Effective pediatric rehabilitation hinges on seamless interdisciplinary collaboration and continuity of care across healthcare settings, educational institutions, and community resources. Coordinated efforts among healthcare professionals, educators, therapists, and social services facilitate comprehensive assessment, goal alignment, and care coordination, ensuring a cohesive and integrated approach to addressing the complex needs of children with neurological disabilities. Furthermore, transitioning from pediatric to adult services necessitates proactive planning and support to facilitate continuity of care and optimize long-term outcomes [21].

This case report underscores the transformative impact of pediatric rehabilitation on enhancing functional outcomes and quality of life for children with complex neurodevelopmental conditions. Through a holistic, family-centered approach encompassing interdisciplinary collaboration, innovative interventions, and ongoing support, pediatric rehabilitation holds immense potential in unlocking the full potential of children with neurological impairments, empowering them to thrive and participate fully in society. Continued research, advocacy, and investment in pediatric rehabilitation are imperative to address existing gaps, improve access to services, and optimize outcomes for this vulnerable population.

## Conclusions

In conclusion, this case report highlights the profound impact of pediatric rehabilitation on a five-year-old child diagnosed with spastic quadriplegia secondary to seizure disorder and global developmental delay. Through a comprehensive and tailored rehabilitation program encompassing multidisciplinary interventions, the child experienced remarkable improvements in motor function, communication skills, and activities of daily living. These gains not only enhance the child's functional independence and quality of life but also alleviate caregiver burden and foster positive family dynamics. In essence, this case report serves as a compelling testament to the transformative power of pediatric rehabilitation, emphasizing its pivotal role in promoting functional independence, enhancing the quality of life, and unlocking the potential of children with complex neurodevelopmental conditions. As we continue to advance our understanding and practice of pediatric rehabilitation, it is imperative to prioritize holistic, evidence-based approaches that prioritize the unique needs and aspirations of each child, ensuring they receive the support and opportunities they deserve to thrive.
